# GFD analysis for BRE zeolite graph through reverse degree and reverse neighborhood degree based topological descriptors

**DOI:** 10.1038/s41598-026-45013-2

**Published:** 2026-04-06

**Authors:** K. Yogalakshmi, D. Easwaramoorthy, G. Muhiuddin, Nabilah Abughazalah, Vladimir Kulish

**Affiliations:** 1 Department of Mathematics, School of Advanced Sciences, Vellore Institute of Technology, Vellore, Tamil Nadu India; 2 Department of Mathematics, Faculty of Science, University of Tabuk, Tabuk, 71491 Saudi Arabia; 3 Department of Mathematical Sciences, College of Science, Princess Nourah bint Abdulrahman University, P.O. Box 84428, Riyadh-11671, Saudi Arabia; 4 Department of Computer Science & Department of Mathematics, Faculty of Science, University of South Bohemia in Ceske Budejovice, Branisovska 1760, CZ-37005 Ceske Budejovice, Czech Republic; 5 Madanapalle Institute of Technology & Science - Deemed to be University, Madanapalle, 517325 Andhra Pradesh India

**Keywords:** GFD analysis, Topological descriptors, Reverse degree, Reverse neighborhood degree, BRE zeolite, Chemistry, Engineering, Environmental sciences, Materials science, Mathematics and computing

## Abstract

Zeolites are minerals that are hydrated aluminosilicate and have a consistent pore structure. The zeolite family will be enhanced by the topological descriptors of molecular structures, which will also minimize the high labor costs involved in generating novel structures and lessen the environmental problems related to the industrial mass production of zeolites. The synthesis of the Brewsterite zeolite (BRE zeolite) framework was created by its significant physicochemical properties. The complex structure of BRE zeolite is characterized by reverse based topological descriptors, which can assist researchers in creating new methods for examining the structure. By employing the Renyi entropy and Generalized Fractal Dimensions (GFD) method based on reverse degree and reverse neighborhood degree based topological descriptors, BRE helps prototype and understand the elemental transformations of zeolites and their inherent self-similarity and complexity properties. Additionally, we have examined the accuracy of linear regression and cubic regression models between the acquired reverse degree and reverse neighborhood degree type Renyi entropy and generalized fractal dimensions of BRE zeolite. Therefore, to investigate the properties of large-format BRE zeolite, the expected linear and cubic correlation analysis reveals a significant correlation between Renyi entropy and GFD.

## Introduction

Fractal theory is a fascinating branch of mathematics that studies complex, self-replicating forms in systems such as nature. Fundamentally, fractal theory analyzes objects that repeat their structure at various scales regardless of how much you zoom in or out. This phenomenon is known as self-similarity. In the 1970 s, mathematician Benoit B. Mandelbrot popularized this concept by recognizing the fractal character of many natural phenomena, including mountains, clouds, and coastlines^1–3^. Fractals frequently have non-integer dimensions, which makes them ideally suited to depict irregular, detailed structures. This contrasts with traditional geometry, where shapes have definite dimensions (such as a line being 1D, a square being 2D, or a cube being 3D). Moreover, the Hausdorff dimension of a fractal strictly exceeds its topological dimension, according to Benoit Mandelbrot’s 1975 formal definition of the term. The Latin word “fractus”, meaning fractured or broken, is the source of the phrase “fractal”. Fractals are usually produced by iterative methods, in which a basic rule is repeatedly used to produce increasingly intricate structures. The fundamental linkages between mathematics, geometry, and the real world are revealed via the study of fractals, particularly through mathematical sets like the Julia and Mandelbrot sets. Applications of fractal theory extend beyond mathematics to the fields of chemical structures, computer graphics, art, medicine, and even the study of natural events. It offers important insights into how basic rules can give rise to complex patterns^1–7^.

A more sophisticated explanation of irregular and self-similar patterns in nature is provided by multifractal theory. It extends classical fractal theory within the study of complex systems^8^. Multifractals allow different parts of a system to exhibit varying levels of complexity and scaling behaviors, unlike classical fractals, which are defined by a single, uniform fractal dimension that characterizes the complexity of the whole structure. Taking into consideration the different scaling exponents found in various system components, this multifractal theory offers a way to comprehend complex systems that are not entirely explicable by a single fractal dimension. A concept termed Generalized Fractal Dimensions (GFD) or Renyi Fractal Dimensions is used in multifractal theory. Also, multifractal theory essentially investigates how various fractal dimensions can coexist inside a single structure, providing a more thorough and nuanced explanation of phenomena like financial markets, turbulent fluids, and galaxy dispersion. Scientists and mathematicians can gain a better understanding of how complexity develops in systems having a variety of frequently chaotic characteristics by examining these multifractal formations. Applications of this theory can be found in disciplines like chemistry, geology, economics, and physics, where the ability to represent real-world systems depends on the variation in scaling behavior^9–11^.

The acronym BRE stands for brewsterite zeolite, which possesses a well-defined framework structure. Perrotta and Smith (1964) first identified the crystal structure of brewsterite, which was further improved to supply information for assessing models on the crystal chemistry of zeolites. The composition $\textrm{K}_{0.02}\textrm{Ba}_{0.48}\textrm{Sr}_{1.42}\textrm{Al}_{4.12}\textrm{Si}_{11.95}\textrm{O}_{32}.n\textrm{H}_{2}\textrm{O}$ was determined by electron microprobe analysis. Due to unquantifiable inaccuracies in the correction factors referred to feldspar standards (ARL microprobe, GLAB program), this does not provide an exact valence balance. The formula $\textrm{K}_{0.02}\textrm{Ba}_{0.50}\textrm{Sr}_{1.48}\textrm{Al}_{4.0}\textrm{Si}_{12.0}\textrm{O}_{32}.10\textrm{H}_{2}\textrm{O}$ may represent the most accurate approximation to the chemical composition. According to the composition determined by electron microprobe examination, brewsterite has a full occupancy of two cation and ten water sites $(\textrm{K}_{0.01}\textrm{Ba}_{0.24}\textrm{Sr}_{0.71})_2-\textrm{Al}_{4.1}\textrm{Si}_{11.9}\textrm{O}_{32}.n\textrm{H}_{2}\textrm{O}$, $a = 6.793 \, (2)$, $b = 17.573 \, (6)$, $c = 7.759 \, (2) \, (Angstrom)$, $\beta = 94.54 \, (3)^{\circ }$, $P2_1/m$. Five water molecules at $2.63-2.83 \, (Angstrom)$ and four framework O atoms at 2.83 and $2.89 \, (Angstrom)$ are bound to the Sr, Ba atom. Sr and Ba alternate with pairs of water molecules at 2.98 or $3.07 \, (Angstrom)$ along a and c to create a two-dimensional structure of intersecting chains. Water molecules and framework O atoms are separated by distances that increase from $2.90 \, (Angstrom)$. Although there is no obvious distinct model, hydrogen bonding is likely^14,15^.

The zeolite can adsorb through micropores selectively due to the BRE structure’s design. It can successfully catch molecules with a particular size and shape. This technique makes use of the zeolite’s effective screening and separating capabilities. There are two types of BRE zeolite: silicon-aluminum and pure silicon. Pure silicon BRE zeolites often show excellent thermal and chemical stability since they are primarily made of silicon elements and do not contain aluminum in their structure. Brewsterite is frequently found in volcanic rocks and is usually found in combination with other zeolites like analcime, mordenite, and clinoptilolite. Brewsterite, similar to other zeolites, has a well-defined crystal structure that enables it to exchange and adsorb ions. This makes it useful for a variety of purposes, such as gas separation, water purification, and as a catalyst in different chemical reactions. Despite being less well-known and utilized than more prevalent zeolites, brewsterite is significant in several industrial and scientific settings due to its special qualities^14–17^.

Prabhu et al. computed several distance and degree based topological indices for zeolite LTA structures^42^. Daniel Paul et al. used relativistic topological models that enable the exact evaluation of degree and distance based structural indices through an orthogonal method of partitioning the bonds in the zeolite AST framework^39^. In another study, Jia-Bao Liu et al. formulated general analytical expressions for degree and degree sum based topological indices of zeolite ACO and enhanced the Shannon entropy method to compute corresponding entropy measures, demonstrating improved discriminatory power^38^. Daniel Paul et al. investigated bond-additive molecular descriptors to analyze the topological features of CLO and KFI zeolites, extended their study to entropy-based comparisons, and evaluated chemical reactivity using characteristic polynomial eigenvalues, particularly the HOMO–LUMO gap^40^. Celin Fiona et al. derived hybrid degree-based topological indices and associated entropy measures for the PWN zeolite and used them to develop regression energy models, achieving strong correlations and effective predictive performance^41^.

Topological indices provide an effective method for examining zeolite structures, allowing for the analysis of their geometric and combinatorial features, such as symmetry, ring sizes, and connectivity designs. Also, topological indices offer insight into the complexity of brewsterite and serve as quantitative indicators of zeolite, helping to establish correlations between size, structure, and properties. Topological indices are crucial in QSAR/QSPR studies and are therefore extensively utilized in the analysis of zeolite structures. These studies are based on the inherent relationships between the structures of zeolites and their properties^8,13,18^. Chemical reactive research, predictive toxicology, and computer-aided drug discovery all make substantial use of topological indices. It has been demonstrated that a variety of topological indices, which have been developed over the years, have promising uses in various domains. Numerous topological indices have been discovered to have strong relationships with a range of physicochemical qualities. These include the Sombor index, redefined Zagreb indices, hybrid indices based on Zagreb, harmonic index, and geometric indices established recently^19–21,35–37^.

Based on these motivations, topological descriptors (or indices) are used to investigate the complexities of zeolites and to analyze the structural differences between various phases of BRE zeolite. The Renyi entropy based Generalized Fractal Dimensions (GFD) of a graph is an information-theoretic metric that reveals details about the complexity and organization of a network, such as that of a zeolite. Renyi entropy and GFD based metrics are the most frequently employed graph invariants for assessing the structural properties of zeolite frameworks and materials. Several Renyi entropy based GFD measures have been created, utilizing vertices, edges, vertex degree patterns, vertex reverse degree patterns, eigenvalues, and other network-related information, and their uses in structural chemistry are numerous. In the current study, we discuss the reverse degree, reverse neighborhood degree, and reverse self-powered degree based topological descriptors of BRE zeolites and conduct a comparative analysis of their Renyi entropy and GFD to assess their structural complexity. Additionally, we estimate both linear and cubic regression models based on Renyi entropy and GFD for some topological indices.

## Preliminaries

This section covers reverse degree and reverse neighborhood degree based topological descriptors, Renyi entropy, and generalized fractal dimensions based on Renyi entropy.

### Reverse based topological descriptors

Zeolite is composed of atoms and bonds that possess specific properties, such as atom category, hybridization, charge, and interaction arrangement, which correspond to the vertices and edges, respectively. The ordered pair $(\mathscr {V}(\mathscr {G}), \mathscr {E}(\mathscr {G}))$ is used in this work to formalize a zeolite as a graph $\mathscr {G}$, and the vertex set being $\mathscr {V}(\mathscr {G})=\{v_1,v_2,...,v_f\}$ with $|\mathscr {V}(\mathscr {G})|= x$ and the edge set being $\mathscr {E}(\mathscr {G})=\{e_1,e_2,...,e_j\}$ with $|\mathscr {E}(\mathscr {G})|= y$. The degree of a vertex $v_f \in \mathscr {V}(\mathscr {G})$ in a graph $\mathscr {G}$ refers to the count of vertices that are adjacent to $v_f$ and is denoted as $d(v_f)$. Suppose that $S(v_f) = \{v_g | (v_f, v_g) \in \mathscr {E}(\mathscr {G})\}$ is a set that includes the neighbors of the vertex $v_f$. The neighborhood degree sum vertices of $v_f$ are denoted by $N(v_f) = \sum _{v_h \in S(v_f)} d(v_f)$^8,19,31–34^. In his work on the boiling point of paraffin, Wiener introduced the idea of topological indices for the first time. When he discovered the first topological index, he called it the “Wiener index”. The most popular topological indices in the literature on mathematics and chemistry are Zagreb and Randic^20–22^.

Kulli^23,24^ defines reverse vertex degree $\mathfrak {R}d(v_f)$ as follows: $\mathfrak {R}d(v_f) = 1 + \Delta (\mathscr {G}) - d(v_f)$. The maximum degree of the graph $\mathscr {G}$, which is the highest degree among all the vertices, is expressed as $\Delta (\mathscr {G})$. In this view, the reverse degree based topological indices for the metal-organic networks TM-TCNB were calculated by Zhao et al. in^25^. Inspired by these, we developed the reverse neighborhood degree sum topological indices $\mathfrak {R}Nd(v_f)$ as follows: $\mathfrak {R}Nd(v_f) = 1 + \Delta _N(\mathscr {G}) - Nd(v_f)$, where $\Delta _N(\mathscr {G})$ is the greatest neighborhood degree sum of a graph vertex. Table 1 provides the reverse degree and reverse neighborhood degree formulas for a variety of topological indices.

For more recent studies on the topological indices of chemical graphs based on reverse degrees, see^21,26,27^.

A general equation for defining reverse degree and reverse neighborhood degree based topological descriptors were provided for $\varphi \in \{\mathfrak {R}d, \mathfrak {R}Nd\}$. This formula represents the sum of the descriptor function applied to all the edges of the graph $\mathscr {G}$, as described below^21,28^.

1 $$\begin{aligned} I^{\varphi }(\mathscr {G})= \sum _{(v_f, v_g) \in \mathscr {E}(\mathscr {G})} I^{\varphi }(v_f, v_g). \end{aligned}$$

Likewise, a similar method was presented to generate multiplicative topological descriptors based on reverse degree and reverse neighborhood degree. This formula is defined as the product of the descriptor function applied to all the edges of the graph $\mathscr {G}$^18,26^.

2 $$\begin{aligned} I^{* \varphi }(\mathscr {G})= \prod _{(v_f, v_g) \in \mathscr {E}(\mathscr {G})} I^{\varphi }(v_f, v_g). \end{aligned}$$

where *I* is a non-negative, two-variable operator and the reverse degree type topological descriptors of graph G are indicated by $I_{\varphi }(\mathscr {G})$ and $I^{* \varphi }(\mathscr {G})$.

Consequently, the descriptor structural function based on reverse degree and reverse neighborhood degree $I_{\varphi }(v_f, v_g)$ has the form $I(\varphi (v_f), \varphi (v_g))$, which is defined for specific instances of index values as follows:

- $$ \varphi (v_f)+\varphi (v_g), \text {First Zagreb Index}\, (\mathfrak {R}M_1(\mathscr {G}))$$
- $$ \varphi (v_f).\varphi (v_g), \text {Second Zagreb Index} \, (\mathfrak {R}M_2(\mathscr {G}))$$
- $$(\varphi (v_f)+\varphi (v_g))^2, \text {Hyper Zagreb Index} \, (\mathfrak {R}HM(\mathscr {G}))$$
- $$\frac{1}{\sqrt{\varphi (v_f).\varphi (v_g)}}, \text {Randic Index} \, (\mathfrak {R}R(\mathscr {G}))$$
- $$\frac{2}{\varphi (v_f)+\varphi (v_g)}, \text {Harmonic Index} \, (\mathfrak {R}H(\mathscr {G}))$$
- $$\frac{2 \sqrt{\varphi (v_f).\varphi (v_g)}}{\varphi (v_f)+\varphi (v_g)}, \text {Geometric-Arithmetic Index} \, (\mathfrak {R}GA(\mathscr {G}))$$
- $$\frac{\varphi (v_f).\varphi (v_g)}{\varphi (v_f)+\varphi (v_g)}, \text {Second Redefined Zagreb Index} \, (\mathfrak {R}ReZG_2(\mathscr {G}))$$
- $$(\varphi (v_f).\varphi (v_g)) \times (\varphi (v_f)+\varphi (v_g)), \text {Third Redefined Zagreb Index} \, (\mathfrak {R}ReZG_3(\mathscr {G}))$$

The reverse degree vertex partition divides the total number of edges in the zeolite structure into separate sets according to the reverse degrees of the end vertices. We represent this as

3 $$\begin{aligned} D^{\mathfrak {R}Nd}(s, t) = |\left\{ (v_f, v_g) \in \mathscr {E}(\mathscr {G}) : \left( \mathfrak {R}d(v_f), \mathfrak {R}d(v_g)\right) =(s, t), \forall s, t \ge 1\right\} |. \end{aligned}$$

In the instance of reverse neighborhood degree sum partition, we indicate

4 $$\begin{aligned} D^{\mathfrak {R}Nd}(s, t) = |\left\{ (v_f, v_g) \in \mathscr {E}(\mathscr {G}) : \left( \mathfrak {R}Nd(v_f), \mathfrak {R}Nd(v_g)\right) =(s, t), \forall s, t \ge 1\right\} |. \end{aligned}$$

Let $K^{\mathfrak {R}d}=\{\mathfrak {R}d(v_f) | v_f \in \mathscr {V}(\mathscr {G})\}$ and $K^{\mathfrak {R}Nd}=\{\mathfrak {R}Nd(v_f) | v_f \in \mathscr {V}(\mathscr {G})\}$. Hence, the potential reverse degree and reverse neighborhood degree sum classes of the zeolite are derived from the following sets.

$$\begin{aligned}&E^{\mathfrak {R}d}=\left\{ (s, t) \in K^{\mathfrak {R}d}\times K^{\mathfrak {R}d}| 1 \le s \le t \le \Delta (\mathscr {G})\right\} \\&E^{\mathfrak {R}Nd}=\left\{ (s, t) \in K^{\mathfrak {R}Nd}\times K^{\mathfrak {R}Nd}| 1 \le s \le t \le \Delta _N(\mathscr {G})\right\} \end{aligned}$$

Therefore, the following forms of simplification of Equations (1) and (2) for the reverse degree and reverse neighborhood degree parameters can be obtained.

5 $$\begin{aligned} I^{\mathfrak {R}d}(\mathscr {G})= \sum _{(s, t) \in E^{\mathfrak {R}d}} D^{\mathfrak {R}d}(s, t) \left[ I^{\mathfrak {R}d}(s, t)\right] . \end{aligned}$$

6 $$\begin{aligned} I^{\mathfrak {R}Nd}(\mathscr {G})= \sum _{(s, t) \in E^{\mathfrak {R}Nd}} D^{\mathfrak {R}Nd}(s, t) \left[ I^{\mathfrak {R}Nd}(s, t)\right] . \end{aligned}$$

7 $$\begin{aligned} I^{* \mathfrak {R}d}(\mathscr {G})= \prod _{(s, t) \in E^{\mathfrak {R}d}} \left[ {I^{\mathfrak {R}d}(s, t)}^{I^{\mathfrak {R}d}(s, t)}\right] ^{D^{\mathfrak {R}d}(s, t)}. \end{aligned}$$

8 $$\begin{aligned} I^{* \mathfrak {R}Nd}(\mathscr {G})= \prod _{(s, t) \in E^{\mathfrak {R}Nd}} \left[ {I^{\mathfrak {R}Nd}(s, t)}^{I^{\mathfrak {R}Nd}(s, t)}\right] ^{D^{\mathfrak {R}Nd}(s, t)}. \end{aligned}$$

### Renyi entropy

In information theory, Renyi entropy is important. One of the family of functionals for measuring the diversity, randomness, or unpredictability of a system is the Renyi entropy, which is a generalization of the Shannon entropy. Alfred Renyi was the one who first presented it. Generalized entropy of a given probability distribution is another name for Renyi entropy. For the below probability distribution, the Renyi Entropy of order $q(\ne 1)$, where *q* is a real number, is defined as^8,11^

9 $$\begin{aligned} RE_q=\frac{1}{1-q} ln \left( \sum _{i=1}^{N}p_{i}^{q}\right) . \end{aligned}$$

where the probability of the random variable that accepts the values $x_1, x_2,..., x_N$ are represented by $p_i \in [0, 1]$.

All of the distribution’s Renyi entropies are identical if the probabilities are all the same, meaning that $RE_q = ln N$. In any other case, the entropies decrease as q increases.

### Some specific cases

$$\bullet$$ If $$q=0$$, then

$$\begin{aligned} RE_0 = ln \, N. \end{aligned}$$

This is referred to as the given probability distribution’s *Hartley entropy*.

$\bullet$ As *q* comes closer to 1, it can be demonstrated that $RE_q$ converges to $RE_1$, which is written as

$$\begin{aligned} RE_1= - \sum _{i=1}^{N}p_{i} \, ln \, p_{i}. \end{aligned}$$

This is the well-known discrete probability distribution entropy known as the *Shannon entropy*.

$\bullet$ Renyi entropy occasionally only applies when q = 2,

$$\begin{aligned} RE_2= - ln \left( \sum _{i=1}^{N}p_{i}^{2}\right) . \end{aligned}$$

$\bullet$ Since $q \rightarrow -\infty$, the limit is as

$$\begin{aligned} RE_{-\infty }= - ln \left( min_{i=1,2,...,N}p_{i}\right) , \end{aligned}$$

which, due to its highest value of $RE_q$, is known as *Max-entropy*.

$\bullet$ Likewise, when $q \rightarrow \infty$, the limit is as

$$\begin{aligned} RE_{\infty }= - ln \left( max_{i=1,2,...,N}p_{i}\right) , \end{aligned}$$

and since it is the smallest value of $RE_q$, this is known as *Min-entropy*.

### GFD analysis

A crucial aspect of the suggested approach is the Renyi entropy based multifractal measure, known as Generalized Fractal Dimensions, which is described in this section as a nonlinear measure to study the complex zeolite structures. Since Renyi entropy is a common nonlinear entropy, it is a crucial tool for generalizing the fractal dimension. The most effective technique in non-linearity analysis for differentiating or estimating the complexity of complex systems in the actual world is the multifractal measure, or GFD, which is studied theoretically. The multifractal theory, which is based on GFD, was methodically developed by Grassberger and Hentschel et al. This section explains the GFD method as well as the topological index based GFD approach that was created from the standard GFD method^9–11^.

For a known probability distribution, the Renyi fractal dimensions, also called the Generalized Fractal Dimensions (GFD), of order $q \in (-\infty , \infty )$ can be described as^8,12^

10 $$\begin{aligned} D_q=\lim _{\varepsilon \rightarrow 0} \frac{1}{q-1} \frac{ln \left( \sum _{i=1}^{N}p_{i}^{q}\right) }{ln \,\varepsilon }. \end{aligned}$$

In this case, generalized Renyi entropy is used to define $D_q$, and $\varepsilon$ is the scaling factor.

### Some particular cases

$$\bullet$$ If $$q=0$$, then

$$\begin{aligned} D_0 = - \frac{ln \, N}{ln \, \varepsilon }, \end{aligned}$$

which simply refers to the fractal dimension.

$\bullet$ When *q* approaches 1, $D_q$ converges to $D_1$, which is determined by

$$\begin{aligned} D_1 =\lim _{\varepsilon \rightarrow 0} \frac{\sum _{i=1}^{N}p_{i} \, ln \, p_{i}}{ln \,\varepsilon }. \end{aligned}$$

We refer to this as the information dimension.

$\bullet$ When $q = 2$, the correlation dimension is denoted by $D_q$.

$\bullet$ The limit instances when $q = -\infty$ and $q = \infty$ are as follows:

$$\begin{aligned} D_{-\infty } =\lim _{\varepsilon \rightarrow 0} \frac{ln (p_{min})}{ln \,\varepsilon }, \end{aligned}$$

$$\begin{aligned} D_{\infty } =\lim _{\varepsilon \rightarrow 0} \frac{ln (p_{max})}{ln \,\varepsilon }, \end{aligned}$$

where $$p_{min} =min\{p_1,p_2,...,p_N\}$$ and $$p_{max} =max\{p_1,p_2,...,p_N\}.$$

## Main results on reverse based topological descriptors for BRE zeolite

**Fig. 1:**
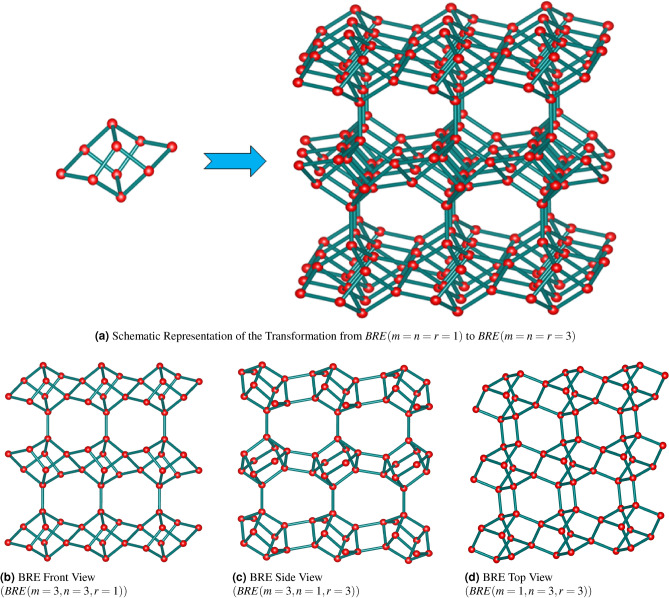
Zeolite Structure of BRE(m,n,r).

The graph-theoretic method can be used to create, compare, and classify zeolite structures. It can also be used to explain the compositions of zeolite structures and predict their properties and changes. In this section, we introduce reverse degree descriptors for the BRE zeolite and use them to calculate the associated information related to Renyi entropy and GFD. The initial structure of BRE zeolite consists of 10 vertices and 14 edges. To form the BRE zeolite structure, this initial framework is extended in three directions and covalently bonded, which can form the BRE zeolite structure by connecting using the initial configuration in a 3D mesh with dimensions $m \times n \times r$, as depicted in Figure 1. Additionally, *BRE*(*m*, *n*, *r*) refers to the brewsterite zeolite where *m*, *n* and *r* denote the number of rows, columns, and layers, respectively, as shown in Figure 1.

Let $\mathscr {G}$ represent the complex framework of *BRE*(*m*, *n*, *r*), for $m,n,r\ge 2$, the total number of vertices and edges of the $\mathscr {G}$ are calculated as $|\mathscr {V}(\mathscr {G})| = 2mr(4n + 1)$ and $|\mathscr {E}(\mathscr {G})| = 16mnr - 2mn - nr +mr$. When $m,n\ge 2$ and $r=1$, the total number of vertices and edges in *BRE*(*m*, *n*, 1) are given by $|\mathscr {V}(\mathscr {G})| = 2m(4n + 1)$ and $|\mathscr {E}(\mathscr {G})| = 14mn-n+m$, respectively.

The reverse degree and reverse neighborhood degree edge partitions of the *BRE*(*m*, *n*, *r*) zeolite molecular graph are determined using Equations (3) and (4). This results in five reverse degree classes and thirty-five reverse neighborhood degree classes for *BRE*(*m*, *n*, *r*) when $m,n,r\ge 2$, as presented in Tables 1 and 3. *BRE*(*m*, *n*, 1) zeolite admits four reverse degree classes for $m,n\ge 2$ and $r=1$, as listed in Table 2.

**Table 1:** Edge partition of *BRE*(*m*, *n*, *r*) with $m,n,r\ge 2$ based on reverse degree $|\mathscr {E}_{\mathfrak {R}d_{f},\mathfrak {R}d_{g}}|$.

$(d_f,d_g)$	$(\mathfrak {R}d_{f},\mathfrak {R}d_{g})$	Frequency
(2,3)	(2,3)	2m(r+1)
(2,4)	(1,3)	2m(r-1)
(3,3)	(2,2)	2(mn+m+2n+r)
(3,4)	(1,2)	2(4mn+2mr+3nr-3m-4n-2r)
(4,4)	(1,1)	16mnr-12mn-7mr-7nr+4m+4n+2r

**Table 2:** Edge partition of *BRE*(*m*, *n*, *r*) with $m,n\ge 2$ and $r=1$ based on reverse degree $|\mathscr {E}_{\mathfrak {R}d_{f},\mathfrak {R}d_{g}}|$.

$(d_f,d_g)$	$(\mathfrak {R}d_{f},\mathfrak {R}d_{g})$	Frequency
(2,3)	(2,3)	4m
(3,3)	(2,2)	2(mn+m+2n+1)
(3,4)	(1,2)	2(4mn-m-n-2)
(4,4)	(1,1)	4mn-3m-3n+2

As described below, the topological descriptor functions are calculated using the following equations and edge partition parameter values, based on the reverse degree, reverse neighborhood degree, multiplicative reverse degree, and multiplicative reverse neighborhood degree, derived from the general Equations (5), (6), (7) and (8).

$$\begin{aligned} \begin{aligned} I^{\mathfrak {R}d}(BRE(m,n,r))=&D^{\mathfrak {R}d}(2, 3) I^{\mathfrak {R}d}(2, 3)+D^{\mathfrak {R}d}(1, 3) I^{\mathfrak {R}d}(1, 3)+D^{\mathfrak {R}d}(2, 2) I^{\mathfrak {R}d}(2, 2)\\&+D^{\mathfrak {R}d}(1, 2) I^{\mathfrak {R}d}(1, 2) +D^{\mathfrak {R}d}(1, 1) I^{\mathfrak {R}d}(1, 1). \end{aligned} \end{aligned}$$

$$\begin{aligned} \begin{aligned} I^{\mathfrak {R}d}(BRE(m,n,r=1))=&D^{\mathfrak {R}d}(2, 3) I^{\mathfrak {R}d}(2, 3)+D^{\mathfrak {R}d}(2, 2) I^{\mathfrak {R}d}(2, 2) +D^{\mathfrak {R}d}(1, 2) I^{\mathfrak {R}d}(1, 2) +D^{\mathfrak {R}d}(1, 1) I^{\mathfrak {R}d}(1, 1). \end{aligned} \end{aligned}$$

$$\begin{aligned} \begin{aligned} I^{\mathfrak {R}Nd}(BRE(m,n,r))=&D^{\mathfrak {R}Nd}(9, 11) I^{\mathfrak {R}Nd}(9, 11)+D^{\mathfrak {R}Nd}(8, 11) I^{\mathfrak {R}Nd}(8, 11)+D^{\mathfrak {R}Nd}(7, 11) I^{\mathfrak {R}Nd}(7, 11)\\&+D^{\mathfrak {R}Nd}(9, 10) I^{\mathfrak {R}Nd}(9, 10)+D^{\mathfrak {R}Nd}(8, 10) I^{\mathfrak {R}Nd}(8, 10)+D^{\mathfrak {R}Nd}(7, 10) I^{\mathfrak {R}Nd}(7, 10)\\&+D^{\mathfrak {R}Nd}(4, 10) I^{\mathfrak {R}Nd}(4, 10)+D^{\mathfrak {R}Nd}(3, 10) I^{\mathfrak {R}Nd}(3, 10)+D^{\mathfrak {R}Nd}(7, 9) I^{\mathfrak {R}Nd}(7, 9)\\&+D^{\mathfrak {R}Nd}(6, 9) I^{\mathfrak {R}Nd}(6, 9) +D^{\mathfrak {R}Nd}(8, 8) I^{\mathfrak {R}Nd}(8, 8)+D^{\mathfrak {R}Nd}(7, 8) I^{\mathfrak {R}Nd}(7, 8)\\&+D^{\mathfrak {R}Nd}(6, 8) I^{\mathfrak {R}Nd}(6, 8)+D^{\mathfrak {R}Nd}(3, 8) I^{\mathfrak {R}Nd}(3, 8)+D^{\mathfrak {R}Nd}(2, 8) I^{\mathfrak {R}Nd}(2, 8)\\&+D^{\mathfrak {R}Nd}(7, 7) I^{\mathfrak {R}Nd}(7, 7)+D^{\mathfrak {R}Nd}(6, 7) I^{\mathfrak {R}Nd}(6, 7)+D^{\mathfrak {R}Nd}(4, 7) I^{\mathfrak {R}Nd}(4, 7)\\&+D^{\mathfrak {R}Nd}(3, 7) I^{\mathfrak {R}Nd}(3, 7)+D^{\mathfrak {R}Nd}(2, 7) I^{\mathfrak {R}Nd}(2, 7)+D^{\mathfrak {R}Nd}(6, 6) I^{\mathfrak {R}Nd}(6, 6)\\&+D^{\mathfrak {R}Nd}(4, 6) I^{\mathfrak {R}Nd}(4, 6)+D^{\mathfrak {R}Nd}(3, 6) I^{\mathfrak {R}Nd}(3, 6)+D^{\mathfrak {R}Nd}(2, 6) I^{\mathfrak {R}Nd}(2, 6)\\&+D^{\mathfrak {R}Nd}(4, 5) I^{\mathfrak {R}Nd}(4, 5)+D^{\mathfrak {R}Nd}(3, 5) I^{\mathfrak {R}Nd}(3, 5)+D^{\mathfrak {R}Nd}(2, 5) I^{\mathfrak {R}Nd}(2, 5)\\&+D^{\mathfrak {R}Nd}(3, 4) I^{\mathfrak {R}Nd}(3, 4)+D^{\mathfrak {R}Nd}(1, 4) I^{\mathfrak {R}Nd}(1, 4)+D^{\mathfrak {R}Nd}(3, 3) I^{\mathfrak {R}Nd}(3, 3)\\&+D^{\mathfrak {R}Nd}(2, 3) I^{\mathfrak {R}Nd}(2, 3) +D^{\mathfrak {R}Nd}(1, 3) I^{\mathfrak {R}Nd}(1, 3)+D^{\mathfrak {R}Nd}(2, 2) I^{\mathfrak {R}Nd}(2, 2)\\&+D^{\mathfrak {R}Nd}(1, 2) I^{\mathfrak {R}Nd}(1, 2)+D^{\mathfrak {R}Nd}(1, 1) I^{\mathfrak {R}Nd}(1, 1). \end{aligned} \end{aligned}$$

$$\begin{aligned} \begin{aligned} I^{* \mathfrak {R}d}(BRE(m,n,r))=&\left[ {I^{\mathfrak {R}d}(2, 3)}^{I^{\mathfrak {R}d}(2, 3)}\right] ^{D^{\mathfrak {R}d}(2, 3)} \times \left[ {I^{\mathfrak {R}d}(1, 3)}^{I^{\mathfrak {R}d}(1, 3)}\right] ^{D^{\mathfrak {R}d}(1, 3)} \times \left[ {I^{\mathfrak {R}d}(2, 2)}^{I^{\mathfrak {R}d}(2, 2)}\right] ^{D^{\mathfrak {R}d}(2, 2)}\\&\times \left[ {I^{\mathfrak {R}d}(1, 2)}^{I^{\mathfrak {R}d}(1, 2)}\right] ^{D^{\mathfrak {R}d}(1, 2)} \times \left[ {I^{\mathfrak {R}d}(1, 1)}^{I^{\mathfrak {R}d}(1, 1)}\right] ^{D^{\mathfrak {R}d}(1, 1)}. \end{aligned} \end{aligned}$$

**Table 3:** Edge partition of *BRE*(*m*, *n*, *r*) with $m,n,r\ge 2$ based on reverse neighborhood degree $|\mathscr {E}_{\mathfrak {R}Nd_{f},\mathfrak {R}Nd_{g}}|$.

$(Nd_f,Nd_g)$	$(\mathfrak {R}Nd_{f},\mathfrak {R}Nd_{\upsilon })$	Frequency	$(Nd_f,Nd_g)$	$(\mathfrak {R}Nd_{f},\mathfrak {R}Nd_{g})$	Frequency
(6,8)	(9,11)	2	(10,14)	(3,7)	2(m+n+r-3)
(6,9)	(8,11)	2m	(10,15)	(2,7)	2(2mr-2m-3r+5)
(6,10)	(7,11)	2(m-1)	(11,11)	(6,6)	2(m-2)(n-2)
(7,8)	(9,10)	2	(11,13)	(4,6)	2(2n-1)
(7,9)	(8,10)	2(m+r-3)	(11,14)	(3,6)	2(2mn-3n+2)
(7,10)	(7,10)	2(m-1)(r-2)	(11,15)	(2,6)	2(2mn-4m+n+2r-5)
(7,13)	(4,10)	2(r-1)	(12,13)	(4,5)	2(r-2)
(7,14)	(3,10)	2(m-1)(r-1)	(12,14)	(3,5)	2(r-2)
(8,10)	(7,9)	4	(12,15)	(2,5)	2(r-2)(3n-5)
(8,11)	(6,9)	4	(13,14)	(3,4)	2(r-1)
(9,9)	(8,8)	2	(13,16)	(1,4)	2(n+r-2)
(9,10)	(7,8)	2m	(14,14)	(3,3)	2(m-1)
(9,11)	(6,8)	2(m+r-2)	(14,15)	(2,3)	2(mn+mr-m-1)
(9,14)	(3,8)	2(m-1)	(14,16)	(1,3)	2(mn+2mr-2m-2n-2r+3)
(9,15)	(2,8)	2(m+r-2)	(15,15)	(2,2)	2mn+mr+4nr-2m-6n-3r+4
(10,10)	(7,7)	6	(15,16)	(1,2)	2(3mn+4mr+5nr-12m-9n-12r+21)
(10,11)	(6,7)	2(m+4n-10)	(16,16)	(1,1)	16mnr-24mn-22mr-21nr+34m+30n+29r-42
(10,13)	(4,7)	2(n-1)			

As a result, the molecular descriptors for *BRE*(*m*, *n*, *r*) and *BRE*(*m*, *n*, 1) zeolite, based on the reverse degree, reverse neighborhood degree, and multiplicative reverse degree, have been calculated as Theorems 1, 2, 3, and 4, respectively.

### Theorem 1

For the BRE zeolite structure denoted as *BRE*(*m*, *n*, *r*), where $m,n,r\ge 2$, the topological descriptors based on reverse degree can be determined as follows:

1. $$\mathfrak {R}M_1(BRE)= 4(8mnr + 2mn + 4mr + nr),$$
2. $$\mathfrak {R}M_2(BRE) = 16mnr + 12mn + 19mr + 5nr + 6m + 4n + 2r,$$
3. $$\mathfrak {R}HM(G)= 2(32mnr + 28mn + 45mr + 13nr + 6m + 4n + 2r),$$
4. $$\mathfrak {R}R(BRE)= \dfrac{1}{3}(48mnr + (12\sqrt{2} - 33)mn + (6\sqrt{2} + \sqrt{3}(\sqrt{2} + 2) - 21)mr + (9\sqrt{2} - 21)nr$$ $$\quad + (\sqrt{3}(\sqrt{2} - 2) - 9\sqrt{2} + 15)m - (12\sqrt{2} - 18)n - (6\sqrt{2} - 9)r),$$
5. $$\mathfrak {R}H(BRE) = \dfrac{1}{15}(240mnr - 85mn - 38mr - 45nr + 12m + 10n + 5r ),$$
6. $$\mathfrak {R}GA(BRE) = \dfrac{1}{15}(240mnr + (80\sqrt{2} - 150)mn + (40\sqrt{2} + \sqrt{3}(12\sqrt{2} + 15) - 105)mr + (60\sqrt{2} - 105)nr$$ $$\quad + (\sqrt{3}(12\sqrt{2} - 15) - 60\sqrt{2} + 90)m - (80\sqrt{2} - 120)n - (40\sqrt{2} - 60)r ),$$
7. $$\mathfrak {R}ReZG_2(BRE)= \dfrac{1}{30} (240mnr + 40mn + 92mr + 15nr + 27m + 20n + 10r),$$
8. $$\mathfrak {R}ReZG_3(BRE)= 2(16mnr + 28mn + 47mr + 11nr + 20m + 12n + 6r).$$

### Theorem 2

For the BRE zeolite structure denoted as *BRE*(*m*, *n*, *r*), where $m,n\ge 2$ and $r=1$, the topological descriptors based on reverse degree can be determined as follows:

1. $$\mathfrak {R}M_1(BRE)= 4(10mn + 4m + n),$$
2. $$\mathfrak {R}M_2(BRE) = 28mn + 25m + 9n + 2,$$
3. $$\mathfrak {R}HM(G)= 2(60mn + 51m + 17n + 2),$$
4. $$\mathfrak {R}R(BRE)= \dfrac{1}{3}((12\sqrt{2} + 15)mn - (3\sqrt{2} - 2\sqrt{6} + 6)m - (3\sqrt{2} + 3)n - 6\sqrt{2} + 9),$$
5. $$\mathfrak {R}H(BRE) = \dfrac{1}{15}(155mn - 26m - 35n + 5 ),$$
6. $$\mathfrak {R}GA(BRE) = \dfrac{1}{15}((80\sqrt{2} + 90)mn - (20\sqrt{2} - 24\sqrt{6} + 15)m - (20\sqrt{2} - 15)n - 40\sqrt{2} + 60),$$
7. $$\mathfrak {R}ReZG_2(BRE)= \dfrac{1}{30} (280mn + 119m + 35n + 10),$$
8. $$\mathfrak {R}ReZG_3(BRE)= 2(44mn + 67m + 23n + 6).$$

### Theorem 3

For the BRE zeolite structure denoted as *BRE*(*m*, *n*, *r*), where $m,n,r\ge 2$, the topological descriptors based on reverse neighborhood degree can be determined as follows:

1. $$\mathfrak {R}NM_1(BRE)= 2(16mnr + 44mn + 53mr + 23nr - 4n - 2r - 6m),$$
2. $$\mathfrak {R}NM_2(BRE) = 16mnr + 206mn + 278mr + 75nr + 240m + 148n + 133r - 34,$$
3. $$\mathfrak {R}NHM(BRE)= 2(32mnr + 470mn + 677mr + 182nr + 390m + 260n + 211r - 54),$$
4. $$\mathfrak {R}NR(BRE)= \dfrac{1}{13860}(221760mnr + (50820\sqrt{2} + \sqrt{3}(4620\sqrt{2} + 18480) - 314160)mn$$ $$\quad + (55440\sqrt{2} + \sqrt{3}(4620\sqrt{2} + 18480) + \sqrt{7}(396\sqrt{2}\sqrt{5} + 3960\sqrt{2}) + 924\sqrt{2}\sqrt{3}\sqrt{5} - 297990)mr$$ $$\quad + (8316\sqrt{2}\sqrt{5} + 69300\sqrt{2} - 263340)nr - (\sqrt{5}(924\sqrt{2}\sqrt{3} - 1386) + 166320\sqrt{2} - \sqrt{11}(630\sqrt{2} + 360\sqrt{7})$$ $$\quad + \sqrt{7}(792\sqrt{2}\sqrt{5} + 2970\sqrt{2} - \sqrt{3}(660\sqrt{2} + 1320)) + \sqrt{3}(2310\sqrt{2} + 34650) - 464310)m + (\sqrt{3}(4620\sqrt{2} - 13860)$$ $$\quad - 138600\sqrt{2} - 16632\sqrt{2}\sqrt{5} + \sqrt{7}(\sqrt{3}(2640\sqrt{2} + 1320) + 1980) + 378840)n - (166320\sqrt{2} + 2310\sqrt{3}$$ $$\quad + \sqrt{7}(396\sqrt{2}\sqrt{5} + 5940\sqrt{2} - 1320\sqrt{3}) + \sqrt{5}(12474\sqrt{2} + \sqrt{3}(924\sqrt{2} - 1848) - 4158) - 401940)r$$ $$\quad + 300300\sqrt{2} + \sqrt{5}(27258\sqrt{2} + \sqrt{3}(924\sqrt{2} - 3696) - 9702) + \sqrt{7}(792\sqrt{2}\sqrt{5} + 9900\sqrt{2} - \sqrt{3}(6600\sqrt{2} + 3960) + 660)$$ $$\quad - \sqrt{11}(360\sqrt{7} - 840) - \sqrt{3}(6160\sqrt{2} + 4620) - 571395),$$
5. $$\mathfrak {R}NH(BRE) = \dfrac{1}{58198140}(931170240mnr - 871678808mn - 694582658mr - 618008820nr + 768885364m$$ $$\quad + 678929850n + 563128764r - 668649981),$$
6. $$\mathfrak {R}NGA(BRE) = \dfrac{1}{29099070}(465585120mnr + (193993800\sqrt{2} + \sqrt{3}(23279256\sqrt{2} + 87297210) - 581981400)mn$$ $$\quad + (155195040\sqrt{2} + \sqrt{3}(23279256\sqrt{2} + 58198140) + \sqrt{7}(6846840\sqrt{2}\sqrt{5} + 25865840\sqrt{2}) + 8953560\sqrt{2}\sqrt{3}\sqrt{5}$$ $$\quad - 611080470)mr + (49884120\sqrt{2}\sqrt{5} + 193993800\sqrt{2} - 494684190)nr - (\sqrt{5}(8953560\sqrt{2}\sqrt{3} - 25865840)$$ $$\quad + 465585120\sqrt{2} - \sqrt{11}(12252240\sqrt{2} + 6466460\sqrt{7}) + \sqrt{7}(13693680\sqrt{2}\sqrt{5} + 10346336\sqrt{2} - \sqrt{3}(8953560\sqrt{2}$$ $$\quad + 11639628)) + \sqrt{3}(2116296\sqrt{2} + 141338340) - 919530612)m + (\sqrt{3}(46558512\sqrt{2} - 29099070)$$ $$\quad - 465585120\sqrt{2} - 99768240\sqrt{2}\sqrt{5} + \sqrt{7}(\sqrt{3}(35814240\sqrt{2} + 11639628) + 21162960) + 628539912)n$$ $$\quad - (465585120\sqrt{2} - 66512160\sqrt{3} + \sqrt{7}(6846840\sqrt{2}\sqrt{5} + 38798760\sqrt{2} - 11639628\sqrt{3}) + \sqrt{5}(66512160\sqrt{2}$$ $$\quad + \sqrt{3}(8953560\sqrt{2} - 14549535) - 51731680) - 849692844)r + 892371480\sqrt{2} + \sqrt{5}(168030720\sqrt{2}$$ $$\quad + \sqrt{3}(8953560\sqrt{2} - 29099070) - 129329200) - \sqrt{11}(6466460\sqrt{7} - 17459442) + \sqrt{7}(13693680\sqrt{2}\sqrt{5} + 64664600\sqrt{2}$$ $$\quad - \sqrt{3}(89535600\sqrt{2} + 34918884) + 22485645) - \sqrt{3}(21162960\sqrt{2} + 157966380) - 884611728),$$
7. $$\mathfrak {R}NReZG_2(BRE)= \dfrac{1}{58198140}(465585120mnr + 1041746706mn + 1152685996mr + 508540890nr + 264707932m$$ $$\quad + 127698696n + 239404275r - 273621049),$$
8. $$\mathfrak {R}NReZG_3(G)= 2(16mnr + 1000mn + 1896mr + 251nr + 2866m + 1432n + 1239r + 592).$$

### Theorem 4

For the BRE zeolite structure denoted as *BRE*(*m*, *n*, *r*), where $m,n,r\ge 2$, the topological descriptors based on multiplicative reverse degree can be determined as follows:

1. $$\mathfrak {R}M_1(BRE)= 3125^{D^{\mathfrak {R}d}(2, 3)} \times 256^{D^{\mathfrak {R}d}(1, 3)}\times 256^{D^{\mathfrak {R}d}(2, 2)} \times 27^{D^{\mathfrak {R}d}(1, 2)} \times 4^{D^{\mathfrak {R}d}(1, 1)},$$
2. $$\mathfrak {R}M_2(BRE) = 46656^{D^{\mathfrak {R}d}(2, 3)} \times 27^{D^{\mathfrak {R}d}(1, 3)}\times 256^{D^{\mathfrak {R}d}(2, 2)}\times 4^{D^{\mathfrak {R}d}(1, 2)},$$
3. $$\mathfrak {R}HM(BRE)= 88817841970012530010487453583933440^{D^{\mathfrak {R}d}(2, 3)} \times 18446744073709551616^{D^{\mathfrak {R}d}(1, 3)}$$ $$\quad \times 18446744073709551616^{D^{\mathfrak {R}d}(2, 2)}\times 387420489^{D^{\mathfrak {R}d}(1, 2)} \times 256^{D^{\mathfrak {R}d}(1, 1)},$$
4. $$\mathfrak {R}R(BRE)= (390508353708945/562949953421312)^{D^{\mathfrak {R}d}(2, 3)} \times (3279644209856305/4503599627370496)^{D^{\mathfrak {R}d}(1, 3)}$$ $$\quad \times (\sqrt{2}/2)^{D^{\mathfrak {R}d}(2, 2)} \times (7049520771918119/9007199254740992)^{D^{\mathfrak {R}d}(1, 2)},$$
5. $$\mathfrak {R}H(BRE) = (1560823428673649/2251799813685248)^{D^{\mathfrak {R}d}(2, 3)}\times (\sqrt{2}/2)^{D^{\mathfrak {R}d}(1, 3)} \times (\sqrt{2}/2)^{D^{\mathfrak {R}d}(2, 2)}$$ $$\quad \times (1718444878736295/2251799813685248)^{D^{\mathfrak {R}d}(1, 2)},$$
6. $$\mathfrak {R}GA(BRE) = (551803563731313/562949953421312)^{D^{\mathfrak {R}d}(2, 3)} \times (7952244240952711/9007199254740992)^{D^{\mathfrak {R}d}(1, 3)}$$ $$\quad \times (4260359467338073/4503599627370496)^{D^{\mathfrak {R}d}(1, 2)},$$
7. $$\mathfrak {R}ReZG_2(BRE)= (5605021331746289/4503599627370496)^{D^{\mathfrak {R}d}(2, 3)} \times (113424204950251/140737488355328)^{D^{\mathfrak {R}d}(1, 3)}$$ $$\quad \times (1718444878736295/2251799813685248)^{D^{\mathfrak {R}d}(1, 2)}\times (\sqrt{2}/2)^{D^{\mathfrak {R}d}(1, 1)},$$
8. $$\mathfrak {R}ReZG_3(BRE)= 205891132094648990023782374475305522563121152^{D^{\mathfrak {R}d}(2, 3)}$$ $$\quad \times 8916100448256^{D^{\mathfrak {R}d}(1, 3)}\times 18446744073709551616^{D^{\mathfrak {R}d}(2, 2)}\times 46656^{D^{\mathfrak {R}d}(1, 2)} \times 4^{D^{\mathfrak {R}d}(1, 1)}.$$

## Analysis of Renyi entropy and GFD based on the reverse degree and reverse neighborhood degree of BRE zeolite

In this section, we utilize the calculated reverse degree and reverse neighborhood degree based topological descriptors to formulate Renyi entropy and GFD measures through Renyi’s method. The concepts of entropy and GFD are structural information that depend on the vertex orbit of information and quantify the intricate structure of graphs. Although there are a number of ways to compute probabilistic entropy, we performed our calculations using Renyi’s model since it is the most often used. In information theory, the entropy of a discrete random variable quantifies the average amount of information or unpredictability embedded in its possible outcomes.

In the BRE zeolite structure, let *N* be the number of distinct self-similar crystallographic orbits, and let $\varepsilon$ be the scaling factor. In order to estimate the Renyi entropy and GFD of the BRE zeolite structure, the probability function for interpreting generalized fractal dimensions with Renyi entropy is modified to $p_{(s,t)}$, which is determined in accordance with the topological index:

$$p_{(s,t)} =\frac{|D^{\varphi }(s, t)| I^{\varphi }(s, t)}{I^{\varphi }(\mathscr {G})}, \, where \, \varphi \in \{\mathfrak {R}d, \mathfrak {R}Nd\}.$$

For BRE zeolite structure, Equation (9) is modified to better represent its structural characteristics. The edges of the zeolite structure are treated as elements, and each edge is given a probability value based on topological indices. The Renyi entropy of reverse degree and reverse neighborhood degree based descriptors $RE_q^{\varphi }(\mathscr {G})$ is defined as:

11 $$\begin{aligned} RE_q^{\varphi }(\mathscr {G})=\frac{1}{1-q} ln \left( \sum _{(s, t) \in \mathscr {E}(\mathscr {G})}p_{(s, t)}^{q}\right) . \end{aligned}$$

The Renyi entropy based GFD is calculated for a complex BRE zeolite structure using topological descriptors that depend on reverse degree and reverse neighborhood degree. Consequently, the formula for the generalized fractal dimensions based on reverse topological descriptors, represented as $D_q^{\varphi }(\mathscr {G})$, which is modified from Equation (10) as follows.

12 $$\begin{aligned} D_q^{\varphi }(\mathscr {G})=\lim _{\varepsilon \rightarrow 0} \frac{1}{q-1} \frac{ln \left( \sum _{(s, t) \in \mathscr {E}(\mathscr {G})}p_{(s, t)}^{q}\right) }{ln \,\varepsilon }. \end{aligned}$$

Investigation of the evolution of intricacy during physicochemical reactions that change mesoporous phases is made possible by numerical estimations of zeolite structure complexity. Thermodynamic stability and structural complexity are anticipated to correlate because the majority of zeolites are metastable phases in their chemical structure platforms^29^. Because structural complexity depends on several chemical and structural aspects including atomic ordering, distortions, modulation techniques, etc., as well as framework topology, it is more complex than topological complexity^30^. The informational complexity of BRE zeolite can be quantified using the suggested parameters. Consequently, it was possible to classify zeolite frameworks based on their topological complexity and examine how they changed into structurally linked microporous structures. To fully understand the impact of structural complexity on zeolite crystallization, consistency, and modifications, more research is required. On the other hand, we can examine the evolution of the systems in terms of both structural information and thermodynamic properties when we apply Renyi entropy and GFD to zeolites.

**Table 4:** Renyi entropy values for reverse degree and reverse neighborhood degree of *BRE*(*m*, *n*, *r*) zeolite, when $m=n=r$.

$RE_q^{\varphi }(BRE(m,n,r))$		Reverse Degree$(\mathfrak {R}d)$					Reverse Neighborhood Degree$(\mathfrak {R}Nd)$				
		q=2	q=3	q=4	q=5	q=6	q=2	q=3	q=4	q=5	q=6
$M_1(BRE)$	m=n=r=2	1.4278	1.3975	1.3778	1.3633	1.3519	3.1355	3.0662	3.0149	2.9751	2.9432
	m=n=r=3	1.2472	1.1643	1.1115	1.0755	1.0493	3.2755	3.1957	3.1361	3.089	3.0504
	m=n=r=4	1.0345	0.91901	0.853	0.81147	0.78337	3.0593	2.9194	2.8198	2.7463	2.6904
	m=n=r=5	0.87033	0.74734	0.68316	0.64529	0.62087	2.8005	2.5834	2.4314	2.3252	2.2498
	m=n=r=6	0.74715	0.62674	0.56801	0.5348	0.51395	2.5508	2.2738	2.098	1.9866	1.9131
$M_2(BRE)$	m=n=r=2	1.3973	1.3572	1.329	1.307	1.2889	2.8833	2.7818	2.7086	2.6515	2.6054
	m=n=r=3	1.4381	1.4084	1.3885	1.3733	1.3611	3.1963	3.0991	3.0272	2.9716	2.9276
	m=n=r=4	1.3072	1.2283	1.1726	1.1322	1.1021	3.1799	3.0741	3	2.9469	2.9077
	m=n=r=5	1.1581	1.0443	0.97302	0.92652	0.89468	3.106	2.9877	2.9011	2.8343	2.7814
	m=n=r=6	1.0262	0.89674	0.82347	0.77895	0.74988	3.0203	2.89	2.7921	2.7143	2.6515
*HM*(*BRE*)	m=n=r=2	1.4173	1.3787	1.3534	1.335	1.3207	2.9406	2.8454	2.773	2.7147	2.6668
	m=n=r=3	1.4517	1.4175	1.3938	1.3758	1.3614	3.2261	3.1421	3.0804	3.0323	2.9933
	m=n=r=4	1.3347	1.2609	1.2106	1.1749	1.1486	3.1877	3.0906	3.0253	2.98	2.9473
	m=n=r=5	1.1972	1.0907	1.0238	0.97938	0.94836	3.1041	2.9931	2.917	2.8616	2.8194
	m=n=r=6	1.0719	0.94771	0.87587	0.83115	0.80135	3.0177	2.8976	2.8144	2.7522	2.7033
*R*(*BRE*)	m=n=r=2	1.0477	0.95344	0.89712	0.85978	0.83336	2.5937	2.4456	2.3646	2.3141	2.2793
	m=n=r=3	0.69508	0.58974	0.53679	0.50612	0.4866	1.706	1.4934	1.3891	1.3271	1.2857
	m=n=r=4	0.51546	0.42307	0.38088	0.35786	0.34369	1.2171	1.0193	0.92752	0.87532	0.84206
	m=n=r=5	0.40894	0.32925	0.29498	0.27684	0.26581	0.93829	0.76515	0.68935	0.64809	0.62257
	m=n=r=6	0.33872	0.26932	0.24068	0.22577	0.21676	0.76125	0.61043	0.54731	0.51383	0.49341
*H*(*BRE*)	m=n=r=2	1.0325	0.93469	0.87588	0.83712	0.81002	2.5661	2.4078	2.3214	2.2675	2.2302
	m=n=r=3	0.67413	0.5684	0.51608	0.48615	0.46726	1.6582	1.4449	1.3402	1.2777	1.2361
	m=n=r=4	0.49596	0.40502	0.36411	0.34198	0.32841	1.1683	0.9736	0.88399	0.83345	0.80146
	m=n=r=5	0.39169	0.31407	0.28114	0.2638	0.25328	0.89446	0.72635	0.65352	0.61414	0.58989
	m=n=r=6	0.32349	0.25634	0.22894	0.21474	0.20616	0.72261	0.57744	0.51727	0.48553	0.46621
*GA*(*BRE*)	m=n=r=2	1.2806	1.2215	1.1825	1.1543	1.1326	3.0749	2.9614	2.8728	2.8043	2.7512
	m=n=r=3	0.93267	0.81845	0.75505	0.71597	0.69002	2.5789	2.3392	2.2051	2.1228	2.0677
	m=n=r=4	0.71113	0.5979	0.54231	0.51071	0.49083	2.0086	1.7252	1.5831	1.5003	1.4466
	m=n=r=5	0.57142	0.46875	0.42195	0.39645	0.38076	1.6142	1.3398	1.213	1.1426	1.0984
	m=n=r=6	0.47674	0.38489	0.34508	0.32392	0.31102	1.3409	1.0893	0.97969	0.92068	0.88435
$ReZG_2(BRE)$	m=n=r=2	1.4168	1.3913	1.3758	1.3649	1.3568	3.0933	3.0141	2.9564	2.9117	2.8756
	m=n=r=3	1.2202	1.1316	1.0731	1.0325	1.003	3.2731	3.1929	3.1343	3.089	3.0524
	m=n=r=4	0.99333	0.87211	0.80373	0.76167	0.73387	3.0389	2.8777	2.7593	2.671	2.6044
	m=n=r=5	0.82461	0.69966	0.63618	0.59963	0.57647	2.7431	2.4885	2.3165	2.2024	2.1248
	m=n=r=6	0.70128	0.58184	0.52527	0.49394	0.4745	2.4622	2.1516	1.9677	1.8572	1.7862
$ReZG_3(BRE)$	m=n=r=2	1.2807	1.2226	1.1887	1.1667	1.1511	2.6769	2.5608	2.4739	2.4036	2.346
	m=n=r=3	1.46	1.4334	1.4168	1.4051	1.3963	2.9497	2.813	2.7107	2.6325	2.5727
	m=n=r=4	1.46	1.4334	1.4168	1.4051	1.3963	2.9319	2.7873	2.6938	2.6304	2.5853
	m=n=r=5	1.4289	1.3853	1.3528	1.3267	1.3052	2.8489	2.6711	2.5503	2.4635	2.3987
	m=n=r=6	1.3498	1.2746	1.2187	1.1766	1.1445	2.7539	2.5376	2.3866	2.2799	2.2037

**Fig. 2:**
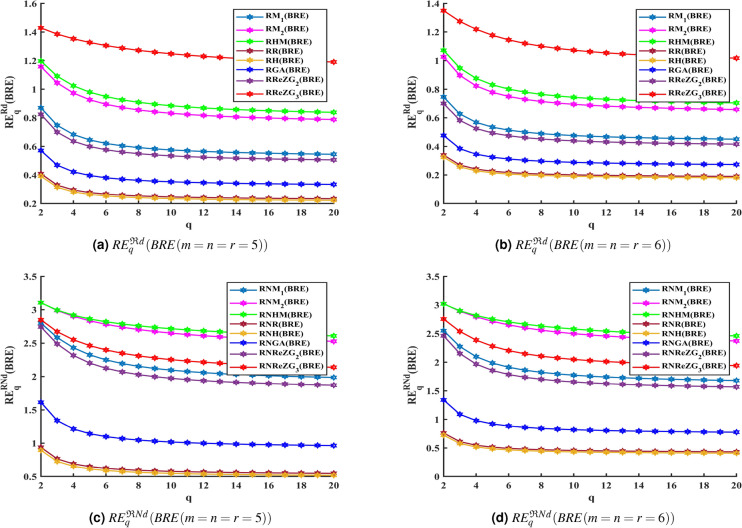
Renyi entropy for BRE zeolite using reverse degree and reverse neighborhood degree based topological indices.

**Fig. 3:**
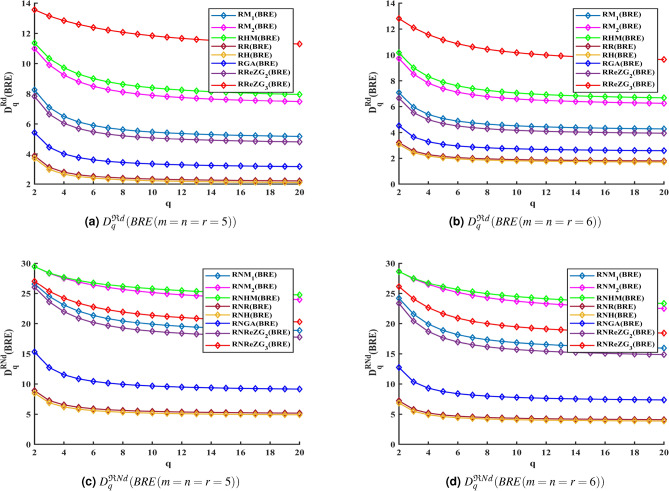
GFD for BRE zeolite using reverse degree and reverse neighborhood degree based topological indices.

By substituting the values of the descriptors from Theorem 1 using Equations (11) and (12), we obtain the reverse degree based Renyi entropy and generalized fractal dimensions of each index. Similarly, the Renyi entropy and GFD values for reverse neighborhood degree based topological descriptors from Theorem 3 can also be derived. For the purpose of comparative analysis, we have focused on cubic BRE zeolite by setting $m = n = r$. Tables 4 and 5 lists the Renyi entropy and GFD measures based on reverse degree and reverse neighborhood degree, which were computed using MATLAB R2022a. In all the descriptors in Tables 4 and 5, excluding the case where $m = n = r = 2$, the values of Renyi entropy and GFD decrease as the iteration number increases, starting from $m = n = r = 3$. Additionally, Figures 2 and 3 present graphical representations of the curves depicting the values obtained from the Renyi entropy and GFD methods, using the general formula of all reverse-based topological descriptors, when reviewed in iterations $BRE(m = n = r = 5)$ and $BRE(m = n = r = 6)$.

According to Figures 2 and 3 and Tables 4 and 5, Renyi entropy and GFD curves corresponding to the reverse degree and reverse neighborhood degree based topological indices of BRE structure may be arranged in the following descending order.

- $$\mathfrak {R}ReZG_3> \mathfrak {R}HM> \mathfrak {R}M_2> \mathfrak {R}M_1> \mathfrak {R}ReZG_2> \mathfrak {R}GA> \mathfrak {R}R> \mathfrak {R}H$$
- $$\mathfrak {R}NHM> \mathfrak {R}NM_2> \mathfrak {R}NReZG_3> \mathfrak {R}NM_1> \mathfrak {R}NReZG_2> \mathfrak {R}NGA> \mathfrak {R}NR> \mathfrak {R}NH$$

**Fig. 4:**
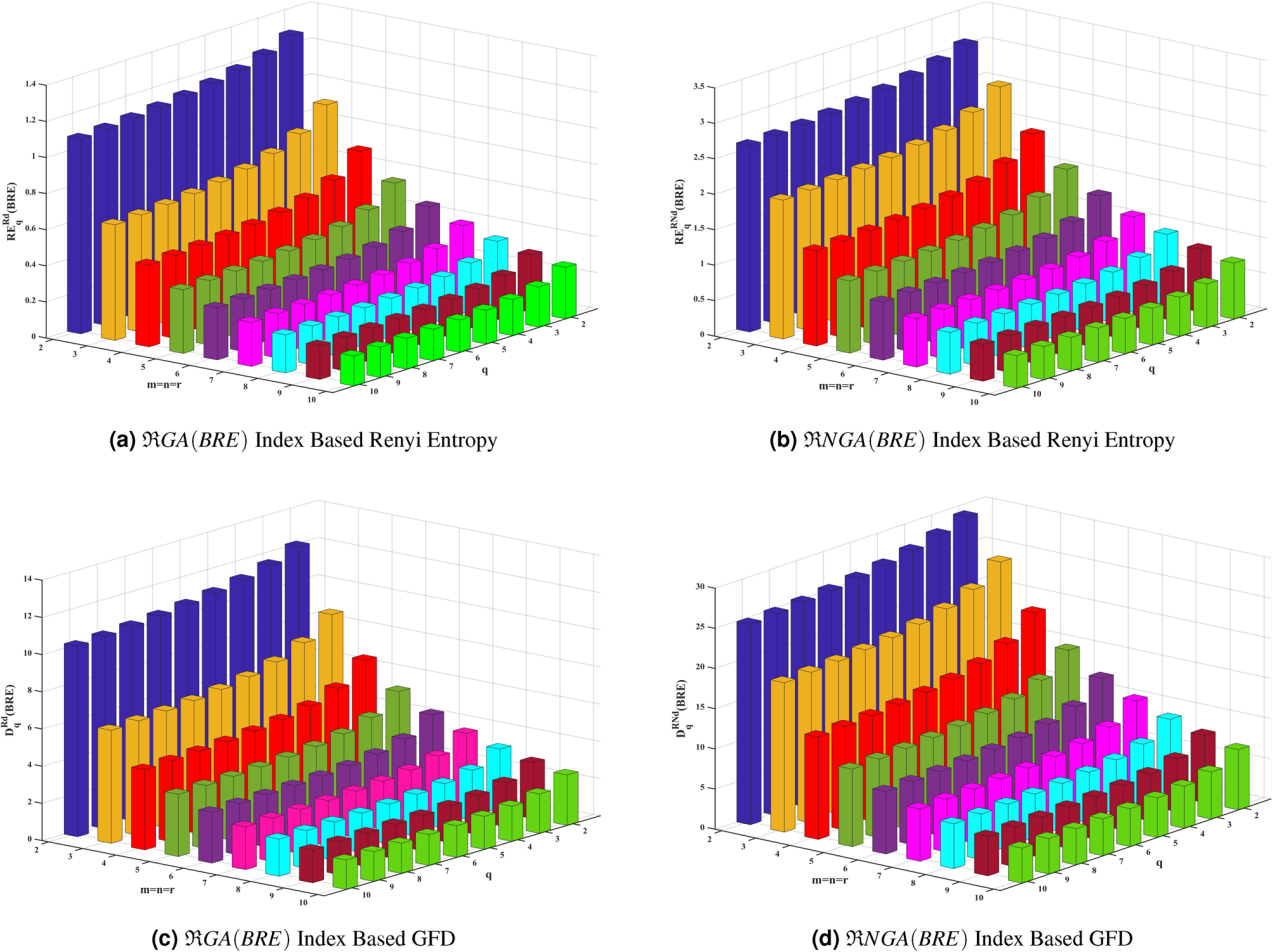
Renyi entropy and GFD for BRE zeolite using reverse topological indices.

In BRE zeolite, as iteration $m = n = r$ and the order *q* increases, resulting in a decrease in the Renyi entropy and generalized fractal dimensions corresponding to the well-known reverse degree and reverse neighborhood degree-based geometric-arithmetic index, as shown in Figure 4. A potent technique for describing zeolite systems, assisting in property prediction, comprehending structural complexities, phase shifts, and structure stability is the combination of topological descriptors, entropy, and GFD measures. The stability and phase transitions of the structures are intimately related to the cubic dimensional system’s information entropy and GFD values. It is determined that reverse degree based measures are lower than reverse neighborhood degree based measures by looking at and evaluating the entropy and GFD values for BRE zeolite.

**Table 5:** GFD values for reverse degree and reverse neighborhood degree of *BRE*(*m*, *n*, *r*) zeolite, when $m=n=r$.

$D_q^{\varphi }(BRE(m,n,r))$		Reverse Degree$(\mathfrak {R}d)$					Reverse Neighborhood Degree$(\mathfrak {R}Nd)$				
		q=2	q=3	q=4	q=5	q=6	q=2	q=3	q=4	q=5	q=6
$M_1(BRE)$	m=n=r=2	13.551	13.264	13.077	12.939	12.831	29.76	29.102	28.615	28.237	27.934
	m=n=r=3	11.837	11.051	10.55	10.207	9.9592	31.088	30.331	29.765	29.319	28.952
	m=n=r=4	9.8182	8.7225	8.096	7.7019	7.4352	29.037	27.708	26.764	26.065	25.535
	m=n=r=5	8.2605	7.0932	6.484	6.1246	5.8928	26.58	24.52	23.077	22.069	21.354
	m=n=r=6	7.0914	5.9486	5.3911	5.0759	4.8781	24.211	21.581	19.912	18.855	18.157
$M_2(BRE)$	m=n=r=2	13.262	12.881	12.614	12.405	12.234	27.366	26.403	25.708	25.166	24.729
	m=n=r=3	13.65	13.368	13.179	13.034	12.918	30.337	29.415	28.732	28.205	27.786
	m=n=r=4	12.407	11.658	11.13	10.746	10.46	30.181	29.177	28.474	27.97	27.597
	m=n=r=5	10.991	9.9116	9.2352	8.7938	8.4916	29.48	28.357	27.535	26.901	26.399
	m=n=r=6	9.7398	8.5112	7.8158	7.3932	7.1173	28.667	27.429	26.5	25.762	25.166
*HM*(*BRE*)	m=n=r=2	13.452	13.085	12.845	12.671	12.535	27.91	27.006	26.32	25.766	25.311
	m=n=r=3	13.778	13.454	13.229	13.058	12.922	30.62	29.822	29.237	28.78	28.41
	m=n=r=4	12.668	11.968	11.49	11.151	10.901	30.256	29.333	28.714	28.284	27.973
	m=n=r=5	11.363	10.352	9.7167	9.2955	9.0011	29.462	28.408	27.686	27.16	26.76
	m=n=r=6	10.174	8.9949	8.3131	7.8887	7.6058	28.642	27.501	26.712	26.122	25.657
*R*(*BRE*)	m=n=r=2	9.9439	9.0493	8.5148	8.1603	7.9096	24.617	23.212	22.443	21.964	21.633
	m=n=r=3	6.5972	5.5973	5.0948	4.8037	4.6185	16.192	14.175	13.185	12.596	12.203
	m=n=r=4	4.8923	4.0155	3.615	3.3965	3.262	11.552	9.6746	8.8033	8.3078	7.9922
	m=n=r=5	3.8813	3.125	2.7998	2.6276	2.5228	8.9055	7.2622	6.5428	6.1512	5.909
	m=n=r=6	3.2148	2.5562	2.2844	2.1428	2.0573	7.2252	5.7937	5.1946	4.8769	4.683
*H*(*BRE*)	m=n=r=2	9.7998	8.8714	8.3132	7.9453	7.6881	24.355	22.853	22.033	21.522	21.167
	m=n=r=3	6.3984	5.3948	4.8982	4.6142	4.4349	15.738	13.714	12.72	12.127	11.732
	m=n=r=4	4.7073	3.8441	3.4559	3.2458	3.117	11.089	9.2406	8.3901	7.9104	7.6068
	m=n=r=5	3.7176	2.9809	2.6684	2.5038	2.4039	8.4896	6.894	6.2027	5.829	5.5987
	m=n=r=6	3.0703	2.433	2.1729	2.0381	1.9567	6.8584	5.4806	4.9096	4.6083	4.4249
*GA*(*BRE*)	m=n=r=2	12.155	11.593	11.223	10.956	10.75	29.184	28.107	27.266	26.616	26.113
	m=n=r=3	8.8522	7.7681	7.1663	6.7955	6.5492	24.477	22.202	20.929	20.148	19.625
	m=n=r=4	6.7495	5.6748	5.1472	4.8473	4.6586	19.064	16.374	15.026	14.24	13.73
	m=n=r=5	5.4235	4.449	4.0048	3.7628	3.6138	15.321	12.716	11.513	10.845	10.425
	m=n=r=6	4.5248	3.6531	3.2753	3.0744	2.952	12.727	10.339	9.2984	8.7384	8.3936
$ReZG_2(BRE)$	m=n=r=2	13.447	13.205	13.058	12.955	12.877	29.359	28.608	28.059	27.636	27.293
	m=n=r=3	11.581	10.74	10.185	9.7994	9.52	31.066	30.304	29.748	29.318	28.971
	m=n=r=4	9.4279	8.2774	7.6283	7.2292	6.9653	28.842	27.313	26.189	25.351	24.719
	m=n=r=5	7.8266	6.6406	6.0381	5.6912	5.4714	26.035	23.619	21.987	20.904	20.167
	m=n=r=6	6.656	5.5224	4.9855	4.6881	4.5036	23.369	20.421	18.676	17.627	16.953
$ReZG_3(BRE)$	m=n=r=2	12.155	11.604	11.283	11.073	10.926	25.407	24.305	23.48	22.813	22.267
	m=n=r=3	13.857	13.605	13.447	13.336	13.253	27.996	26.699	25.728	24.986	24.418
	m=n=r=4	14.046	13.852	13.74	13.664	13.608	27.827	26.455	25.567	24.965	24.538
	m=n=r=5	13.562	13.148	12.839	12.592	12.388	27.039	25.352	24.205	23.381	22.766
	m=n=r=6	12.811	12.097	11.567	11.167	10.863	26.138	24.085	22.652	21.639	20.915

Furthermore, the reverse degree and reverse neighborhood degree based GFD measures are higher than Renyi entropy measures. We use the calculated measure to predict the different potential energies dispersed throughout the BRE zeolite structure via bonds since the entropy and GFD measures explained in the article are obtained using Renyi’s method and are directly related to the probability distribution of the edges within the molecular organization.

The mathematical results align well with the underlying chemistry and structural characteristics of zeolites. Specifically, the descriptors based on reverse degree and reverse neighborhood degree quantify variations in framework branching, local connectivity, and heterogeneity within the zeolite structure. Differences in GFD values are attributed to variations in the multiscale connectivity and structural complexity of the BRE framework, signifying more complex networks and higher topological heterogeneity.

GFD measures are shown to be more effective than Renyi entropy measures, as GFD provides a richer description of multiscale structural complexity rather than simply predictive superiority for a specific physicochemical property. Additionally, by relating entropy and GFD trends to the iteration parameters (*m*, *n*, *r*), it is shown that higher levels of iteration lead to increased structural complexity and qualitatively represent synthesis dependent framework development. These enhancements offer a more precise interpretation of the mathematical results while maintaining alignment with the theoretical framework of the study.

**Fig. 5:**
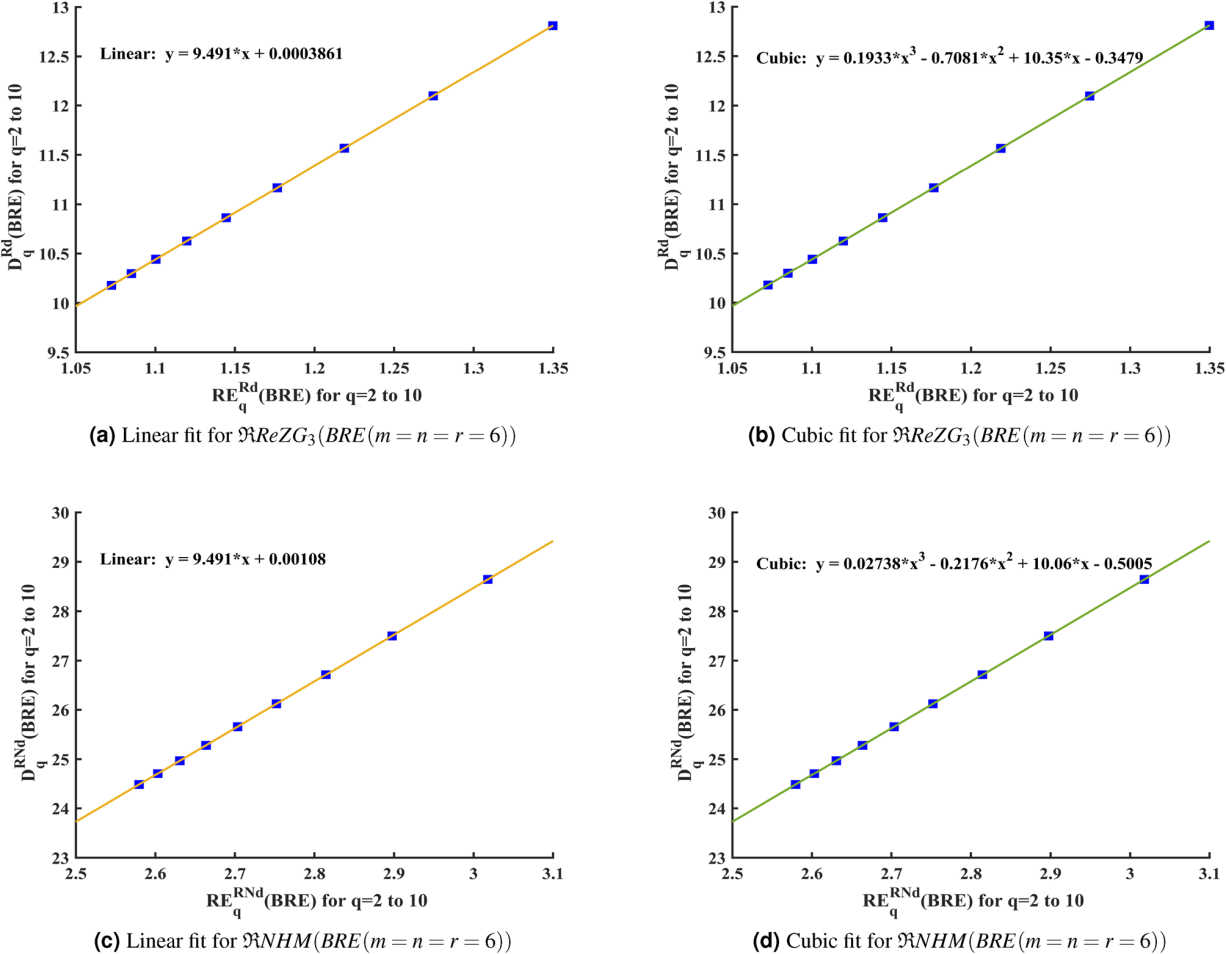
Linear and cubic regression for BRE zeolite.

Figure 5 illustrates scatter plots with both linear and cubic fits for high potential predictive entropy and GFD. The outcomes from Figure 5 suggest that both linear and cubic regression models serve as useful tools for forecasting molecular interactions within the BRE zeolite structure. We suggest the following linear and cubic regression models to correlate the Renyi entropy values, which was produced using the reverse degree based third redefined Zagreb index and reverse neighborhood degree based harmonic index, with the GFD values of BRE zeolite.

13 $$\begin{aligned} y = f_0 * x + c. \end{aligned}$$

14 $$\begin{aligned} y = f_0 * x^3 + f_1 * x^2 + f_2 * x + c. \end{aligned}$$

Here, GFD values, entropy values, and intercept are denoted by *x*, *y*, and *c*, respectively. In contrast, the slopes are $f_0$, $f_1$, and $f_2$.

Equations (13) and (14) are used to obtain the regression equations of BRE zeolite:

$$D_q^{\mathfrak {R}ReZG_3} = 9.491 * x + 0.0003861.$$

$$D_q^{\mathfrak {R}ReZG_3} = 0.1933 * x^3 - 0.7081 * x^2 + 10.35 * x - 0.3479.$$

$$D_q^{\mathfrak {R}NHM} = 9.491 * x + 0.00108.$$

$$D_q^{\mathfrak {R}NHM} = 0.02738 * x^3 - 0.2176 * x^2 + 10.06 * x - 0.5005.$$

Linear and cubic regressions are useful in predicting the relationship between Renyi entropy and GFD of BRE zeolite structure by the provided regression equations and analyze the variations in each iteration of BRE zeolite. Furthermore, the predicted values produced by linear regression and cubic regression equations are more accurate. Reverse degree based third redefined Zagreb index ($\mathfrak {R}ReZG_3$) and reverse neighborhood degree based harmonic index ($\mathfrak {R}NHM$) consistently demonstrated the highest correlation with GFD over all iterations of the BRE zeolite, reflecting their greater sensitivity to structural complexity and justifying their selection as effective predictive indices.

## Conclusion

In this study, we derived the correct analytical expression for the topological descriptors using a graph-theoretical edge partition method, specifically for the reverse degree and reverse neighborhood degree-based topological descriptors of the BRE zeolite. In addition, we identified the multiplicative indices that can be used to calculate the multiplicative general expressions through the edge partition method. Also, we calculated the Renyi entropy and GFD values based on the analytical expression of the reverse-based topological descriptors. The goal of this study is to demonstrate that the GFD measurements for the BRE zeolite outperform the Renyi entropy measurements. Moreover, the GFD metrics for all topological descriptors highlight the complexity of the BRE zeolite at various length scales, as the GFD value decreases with each iteration, providing valuable insights into its hierarchical structure and structural stability. This approach can be utilized to determine how it can assist in QSPR/QSAR research of zeolite crystals. As a possible future direction, this analytical framework approach could be applied to other zeolite families to facilitate systematic comparative analyzes of structural complexity among different structural types. The predictive capacity of the connection between Renyi entropy and GFD information, derived from reverse-based topological descriptors, was examined using linear and cubic regression models. Additionally, the regression study’s findings indicate that Renyi entropy is related to the potential GFD of the BRE zeolite.

## Data Availability

All data generated or analyzed during this study is included in this published article.
